# An Army of 53,000 Always in the Trenches

**Published:** 1920-05-15

**Authors:** 


### THE MOTHERS OF ENGLAND.

### An Army of 53,000 Always in the Trenches.

On Wednesday, May 6, the Council for Promoting the Higher Training of Midwives and for the British Hospital for Mothers and Babies at Woolwich held its annual meeting in the Great Hall of "Bart's." One of the many minor tragedies of the war has been the deferred building of the hospital, containing forty-two beds and capable of nursing some 650 in a year, with out-patient department and treatment for the poor in their own homes. The higher training intended is to cover one year or two years, in order to send out an expert body of women competent to teach others, and to take charge of maternity hospitals and nursing homes.

There can be no doubt that such higher training is needed. When three months was thought a sufficient, and six months a liberal, time of training the actual birth was practically the only matter considered. That a midwife needed to learn the practical care of an infant would have appeared an absurd statement, nor was pre-natal care or observation thought to come into the scope of her work. As to the establishment of lactation, no one supposed that to depend at all upon skill in the nurse. A mother either could or could not, or would or would not, nurse her child herself, and there an end. In the negative case the wet-nurse or the bottle must be resorted to. While if the infant suffered from "sore eyes," perhaps losing its sight in consequence, this was a dispensation of Providence to be borne with resignation. Short courses of three or six months' training cannot fully qualify a woman to deal with the multiplicity of complications possible. And, as was wisely said by Miss Alice Gregory, who is secretary to the hospital, when, after such brief training, a woman works year after year alone in a country district is it not almost certain that she will sink insensibly into the ideas of the people round her on a subject which has scarcely been fully redeemed from barbarism? More than this, knowledge and skill in midwifery and infant care are fast developing, and need be so if our hideous mortality rate is to be lowered. One purpose of the Training Centre will be to provide post-graduate courses to which a trained nurse could come after any three years of work.

Miss Gregory's own experience of conditions in Somerset at a date not very remote bears out the term "barbarism." Often a mother might be heard to hope her trial might not come at Christmas-time, when the local midwife could seldom walk straight. Two full bottles of spirits were essential to the preparations, partly for hospitality, partly for the patient, who would be told she could not get through without plenty of stimulant. Sir Richard Temple compared the mothers of the country to an army, of whom 53,000 were always in the trenches at any given time, each having to go over the top, with a mortality rate of nine daily, apart from the injuries; while to include the young recruits this army must be doubled, and their mortality rate reaches the terrific number of 260 daily. Only the distribution of this force all over the country prevents us from realising the full horror.

It is to be hoped that the expansion of the present hospital will not be long delayed. At present it gives a sound six months' training, at £30 for a three-year trained nurse, or eight months for a one-year trained candidate, or a minimum of one year for £40 to those who have had no training at all. To any willing to sign an agreement to practise as a district midwife the Ministry of Health will give a grant of £20 towards the fees. Pupils are here received between the ages of twenty-one and thirty-five. The work might well, therefore, gain recruits from among the many ex-V.A.D.s in search of a profession. Its service to the community is incalculable, and its opportunities of influence for good are very great.

